# Safety and performance of the novel balloon-in-basket pulsed field ablation system: initial 100-patient experience at a high-volume electrophysiology center

**DOI:** 10.1007/s10840-026-02357-z

**Published:** 2026-06-03

**Authors:** Roland Richard Tilz, Sascha Hatahet, Charlotte Eitel, Sorin Popescu, Roman Mamaev, Kohei Ukita, Mirco Küchler, Suzanne de Waha, Karl-Heinz Kuck, Jan-Per Wenzel

**Affiliations:** 1 Department of Rhythmology, University Heart Center Lübeck, University Hospital Schleswig-Holstein, Campus Lübeck, Lübeck, Germany; 2 German Center for Cardiovascular Research (DZHK), Partner Site Hamburg/Kiel/Lübeck, Lübeck, Germany

**Keywords:** Pulmonary vein isolation, Atrial fibrillation, Pulsed field ablation, Balloon-in-basket, Workflow

## Abstract

**Background:**

The balloon-in-basket(BiB) pulsed field ablation(PFA) system integrates real-time electroanatomic mapping and contact sensing to streamline workflows, but clinical experience remain limited. The aim was to descriptively evaluate procedural performance, acute safety, and feasibility of the BiB-PFA-system in consecutive atrial fibrillation(AF) patients treated at a high-volume electrophysiology center.

**Methods:**

This prospective study included the first 100 consecutive patients undergoing pulmonary vein isolation(PVI) with the BiB-PFA-system between 01/2024 and 10/2025. The initial 30 patients were part of VOLT^TM^-CE-Mark-study and 25 of VOLT^TM^-CE-Mark-extended-study. It included a 20-minute waiting periode, pre-/postablation left-atrial mapping and phrenic nerve pacing; subsequent procedures relied solely on BiB-PFA-system. Additional linear or extra-pulmonary ablations were performed at operator discretion. Follow-up(≥ 6 months) as per institutional standart was available in 52 patients.

**Results:**

Patients were 68[62;76] years old, 41% were female, and 47% had paroxysmal AF. Median procedure duration was 59[48.0;71.0] and BiB-dwell time 26.0[21.0; 37.5] min. For VOLT™-CE-Mark-study patients, procedure and BiB-dwell times were 72.5[67.3;85.0] and 25.0[21.0;28.2] min, compared with 48.5[36.5;59.0] and 23.5[18.3;36.3] min in non-study patients. All PVs were successfully isolated with 100% first-shot success using a median of 4 applications. Additional ablations included posterior wall(41%), anterior line(5%), CTI(3%), and SVC isolation(6%). One groin complication(1%) requirng intervention occured. Same-day discharge was conducted in 8%, limited primarily by institutional policy. Freedom from atrial tachyarrhythmia was 86% at median follow-up of 293[184;364]days.

**Conclusion:**

The BiB-PFA system enabled PVI with consistent first-shot success and a low rate of periprocedural complications. Follow-up available in a subset of patients suggested favorable early rhythm outcomes.

**Take home message:**

•* This study provides the first clinical experience with the BiB-PFA-system, characterizing procedural workflows and acute safety in routine practice*

•* This study reports the first mid-term follow-up data for the BiB-PFA system, demonstrating an 86% freedom from atrial tachyarrhythmia after a 60-day blanking period at a median follow-up of 293 [184-364] days.*

•* The integrated mapping-and-ablation design enabled efficient PVI with consistent first-shot success and short procedure times.*

•* Ablation strategies beyond PVI—including posterior wall ablation, anterior line ablation, CTI ablation, and SVC isolation—were feasible and could be performed safely within the same workflow.*

•* A low rate of periprocedural complications was observed in this cohort, including one vascular complication requiring intervention, supporting a favorable acute safety profile in a high-volume electrophysiology center.*

**Graphical abstract:**

**Figure:**
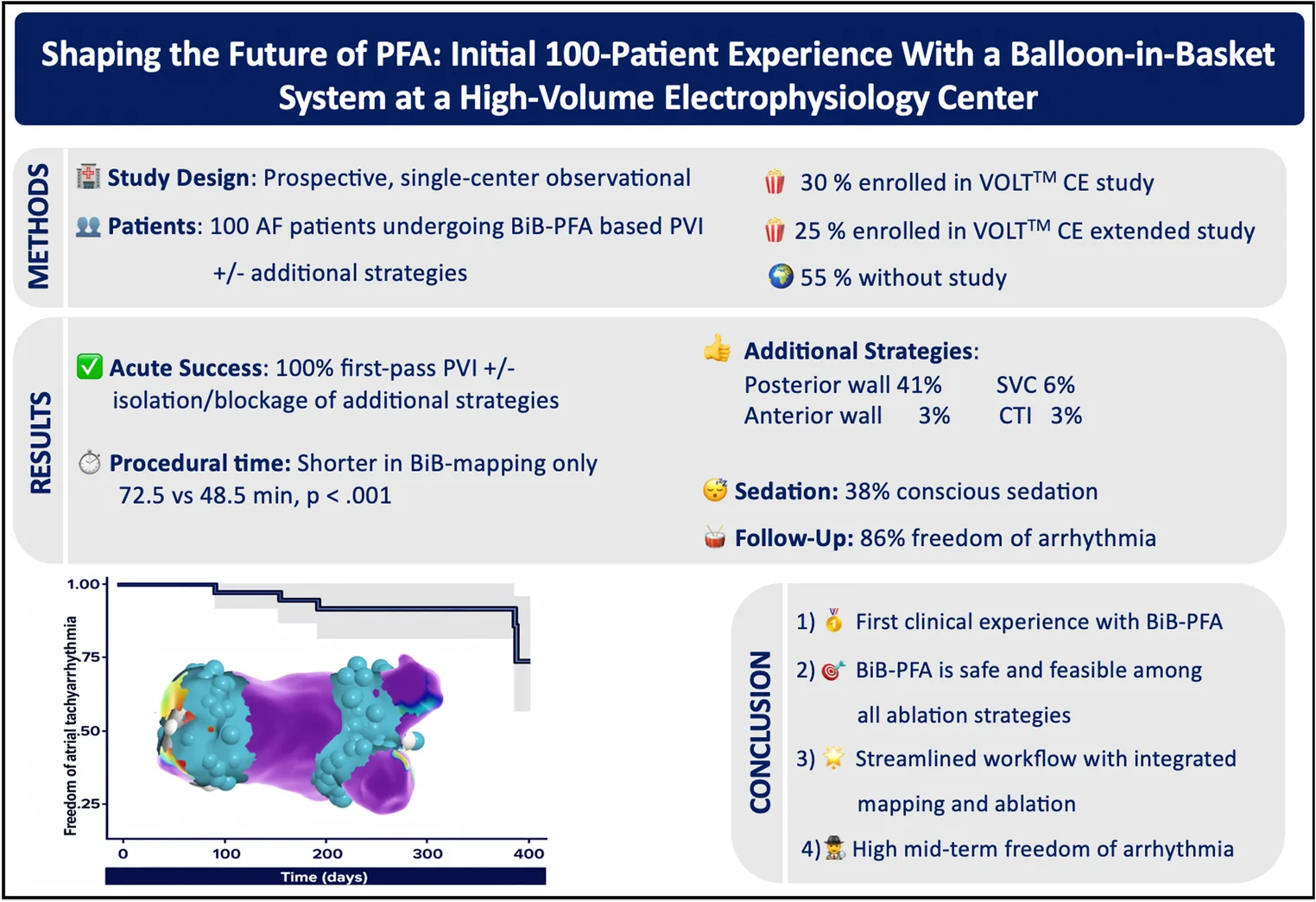

## Introduction

Pulsed-field ablation (PFA) has emerged as a novel, non-thermal energy source for catheter-based treatment of atrial fibrillation (AF), enabling selective myocardial ablation with minimal risk to adjacent structures [1]. The VOLT™-system (Abbott) represents a next-generation PFA catheter that combines a balloon-in-basket (BiB) design with integrated mapping electrodes, enabling both efficient lesion creation and high-resolution mapping within a single system [2]. The expandable basket structure ensures stable, circumferential wall contact during energy delivery, allowing homogeneous lesion formation and potentially reducing shear forces, hemolysis, and inflammatory reactions compared to other PFA or thermal ablation techniques [3–5]. The integration of ablation and mapping into one catheter additionally has the potential to streamline AF ablation workflows and reduce procedural complexity.

Early feasibility studies, including the multicenter VOLT^TM^-CE-Mark and VOLT^TM^-AF-IDE-study, have demonstrated high acute pulmonary vein isolation success rates and favorable procedural characteristics without major periprocedural complications [2, 6]. However, clinical experience with the BiB-PFA-system remains limited. To address this evidence gap, we report the first clinical experience with the BiB-PFA-system, detailing procedural workflows alongside acute safety and efficacy in a high-volume electrophysiology center.

## Methods

### Study population and trial design

This prospective, single-center, non-randomized study enrolled consecutive patients with AF undergoing first-time BiB-PFA. Thirty patients were included in the VOLT™-CE-Mark study, and 25 in the VOLT™-CE-Mark-Extended study [2]. Eligible patients were ≥ 18 years old with documented AF and provided written informed consent. Exclusion criteria included active infection, body mass index ≥ 40, stroke or myocardial infarction within the previous three months. Two patients were excluded from the present analysis: one due to a markedly prolonged procedure duration related to intra-procedural waiting time for operator availability, and one patient who developed pericardial tamponade following transseptal puncture before ablation was performed. The study was approved by the local ethics committee and conducted in accordance with the Declaration of Helsinki [7].

## General procedural management

All patients underwent a standardized preprocedural evaluation according to institutional protocol. In individuals with elevated thromboembolic risk, transesophageal echocardiography was performed to exclude left atrial thrombus. Vitamin K antagonists were continued at a therapeutic INR (2.0–3.0), while direct oral anticoagulants were withheld on the morning of the procedure. Sedation level (conscious versus deep) was determined primarily by patient preference after structured counseling. Non of the patients suffered from anxiety or panic disorder. Deep sedation was carried out using propofol, midazolam, and fentanyl. In conscious sedation, no propofol infusion was administered to maintain partial patient responsiveness. Thereby, multimodal analgesia with metamizole, midazolam, fentanyl, and lidocaine was applied according the AWAKE-protocol previously published by our group [8]. Femoral venous access was obtained via two ultrasound-guided punctures with 8 Fr sheaths, and a diagnostic catheter was positioned in the coronary sinus (CS). In patient number 79, venous access was obtained via the jugular vein due to an inferior vena cava occlusion.

Transseptal puncture was performed using an SL1 sheath (Abbott) and a BRK1 needle under fluoroscopy using the modified Brockenbrough technique. No intracardiac echocardiography was used. After transseptal access, intravenous unfractionated heparin was administered to maintain an activated clotting time ≥ 300 s.

The transseptal sheath was then exchanged for a steerable 13 Fr sheath (Agilis™ NxT, Abbott), and PV access was achieved using a 0.035-inch guidewire or in later cases the wire from the SL1-sheath (Abbott).

Patients enrolled in the VOLT™-CE-Mark (patient 1–30) and VOLT™-CE-Mark-Extended studies (patient 74–94, 96–99) followed the standardized VOLT™-CE protocol, which included pre- and post-ablation phrenic nerve pacing via the superior vena cava, pre- and post-ablation left-atrial mapping, and a 20-minute waiting period. The two study phases differed in their mapping strategy: in the VOLT™-CE-Mark study, left-atrial voltage mapping was performed using a high-density mapping catheter (Advisor™ HD Grid, Abbott), whereas in the VOLT™-CE-Mark-Extended study anatomical reconstruction was performed using the BiB-PFA catheter alone. Posterior wall ablation (PWA) was additionally allowed in the VOLT™-CE-Mark-Extended study. PFA was delivered at 1800 V with at least two rotated applications per vein, allowing a maximum of eight per vein. For right-sided PVs, phrenic nerve function was evaluated using pacing form each spline with an output of 20mV/10ms; if diaphragmatic capture occurred, ablation proceeded at 1400 V with a minimum of three applications.

PWA was performed in patients with persistent af as follows: 1800 V was delivered at with region-specific spline activation to secure adequate wall contact, using a minimum of two energy applications. For perimitral flutter or evidence of anterior wall fibrosis, anterior line (AL) ablation was performed at 1800 V with directed spline activation to maintain consistent wall contact, again with at least two applications. In cases of documented typical atrial flutter, cavotricuspid isthmus (CTI) ablation was performed following catheter repositioning into the right atrium, using selective spline deactivation at 1800 V with at least two applications. No nitroglycerin was administered during CTI ablation. Bidirectional block was confirmed by sequential pacing maneuvers. Superior vena cava (SVC) isolation was performed at the operator’s discretion. After an initial test application train, the full train was delivered only if sinus node integrity was preserved. All splines were activated at 1800 V, with a minimum of two applications administered.

### Postprocedural management

Hemostasis was achieved using vascular closure devices or figure-of-eight sutures with a compression bandage, which was removed after 1–4 h, and sutures were removed the following day. Transthoracic echocardiography was performed routinely immediately post-procedure, at one hour, and on postoperative day one to exclude pericardial effusion. Oral anticoagulation was restarted six hours after ablation and continued for at least two months, with long-term continuation guided by the CHA₂DS₂-VA score according to current guidelines [9, 10]. A class IC or class III antiarrhythmic drug and a proton pump inhibitor were prescribed for two months. Follow-up visits at 3, 6, and 12 months included 12-lead ECG and systematic 24 h Holter monitoring. Arrhythmia recurrence was defined as any atrial tachyarrhythmia ≥ 30 s beyond a 60-day blanking period. For the purpose of this analysis, follow-up assessments from 6 months onward were intentionally selected to provide a robust evaluation of long-term outcomes.

### Intravenous fluid management

Intravenous fluids were administered during the procedure in patients with low blood pressure or clinical signs of hypovolemia. In such cases, up to 500–1,000 ml of balanced full-electrolyte solution were infused to maintain hemodynamic stability.

Post-procedurally, no standardized fluid regimen was applied. However, patients with baseline eGFR < 60 ml/min/1.73 m² received a single infusion of 500–1,000 ml balanced full-electrolyte solution on the first post-procedural day after routine blood sampling. Fluid administration was guided by clinical judgment, renal function, and individual volume status.

### Blood sampling and analysis

Venous blood samples were collected at two time points: (1) after femoral venous access before ablation, and (2) on the morning of the first post-procedural day. All patients were fasting. A comprehensive biomarker panel was analyzed, including inflammatory (leukocytes, platelets, CRP), myocardial (troponin T, CK, myoglobin), renal (creatinine, eGFR), and hemolysis-related markers (LDH, bilirubin, haptoglobin, Hb). Leukocytes and platelets were measured from EDTA whole blood (XN-9000, Sysmex), CRP by immunoturbidimetry (Cobas c503), troponin T and myoglobin by ECLIA (Cobas e801), CK by enzymatic UV photometry (Cobas c702), and LDH, bilirubin, haptoglobin, and creatinine by standard photometric assays (Cobas c503). eGFR was calculated using the CKD-EPI formula. All analyses were performed at the routine central laboratory of University Hospital Schleswig-Holstein, Campus Lübeck.

### Statistical analysis

Continuous data were tested for normality using the Shapiro–Wilk test and are presented as median (Q1;Q3). Between-group comparisons were made using independent-samples t-tests or Mann–Whitney U tests. Categorical variables are reported as absolute and relative frequencies and compared using Chi-square tests. Within-group comparisons used paired t-tests or Wilcoxon signed-rank tests. Correlations between biomarker changes and PFA applications were analyzed using Pearson or Spearman coefficients, based on distribution. All analyses were performed with IBM SPSS Statistics v29.0.1.0 (IBM Corp., Armonk, NY, USA). Two-sided p-values < 0.05 were considered statistically significant.

Three exploratory subgroup analyses were conducted according to procedural metrics:

1. Comparison of PVI-only patients treated within the VOLT™-CE-Mark-study (patient number 1–30) versus non-study patients undergoing PVI-only.
2. Comparison of patients treated within the VOLT™-CE Mark-study (patient number 1–30) versus those enrolled in the VOLT™-CE-Mark-extended cohort(patient number 74–94 and 96–99).
3. Comparison of PVI-only non-study patients receiving deep sedation versus conscious sedation.

For all subgroup analyses, the same statistical methods as described above were applied.

## Results

### Baseline characteristics

**Fig. 1:**
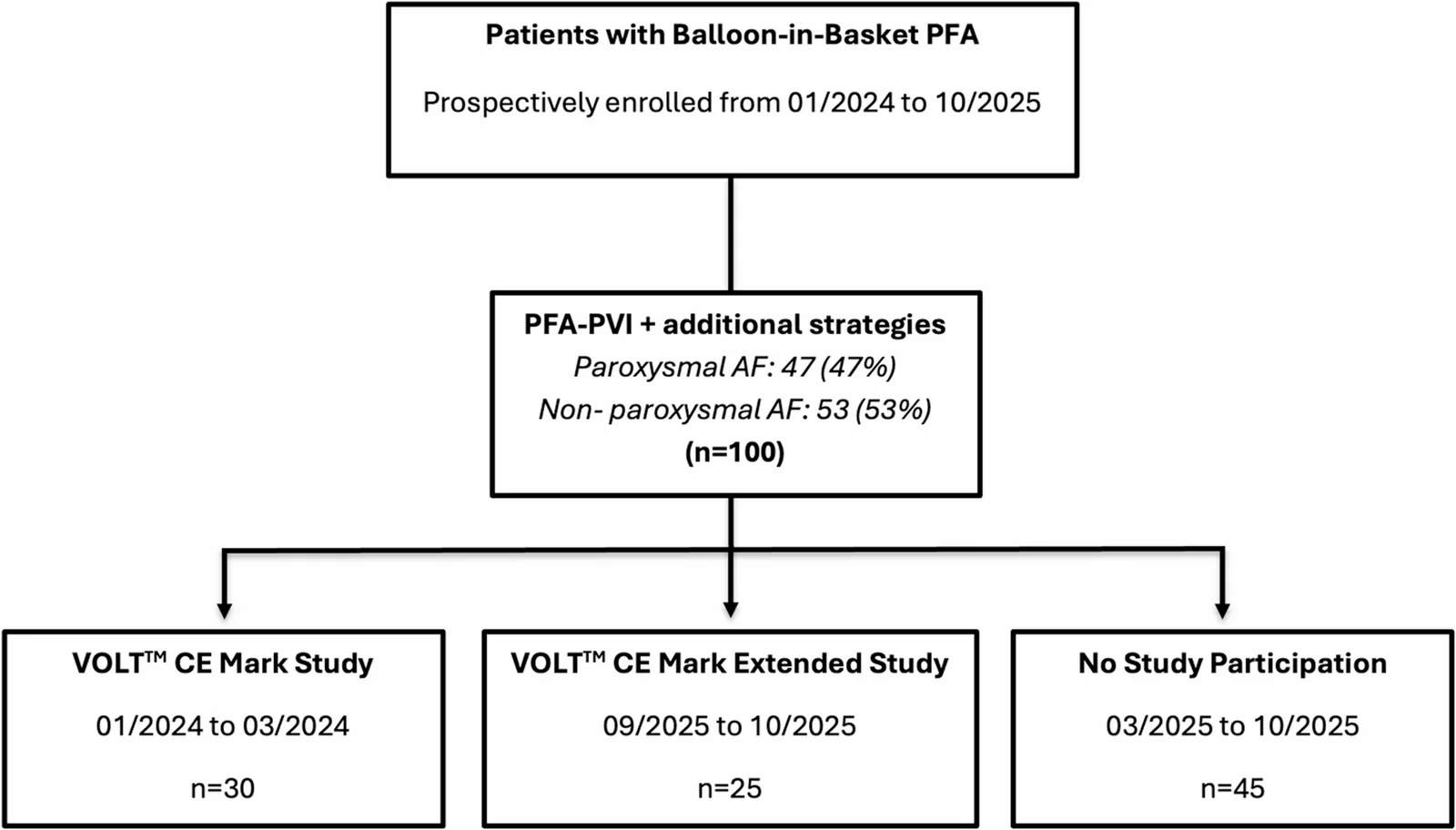
Study PRISMA (STROBE format). Figure 1 represents the study flow chart. AF = atrial fibrillation; PFA = pulsed field ablation; PVI = pulmonary vein isolation

The study population consisted of 100 patients with a median age of 68 [62; 76] years, of whom 41% were female. Paroxysmal AF was present in 47% of patients, and the median CHA₂DS₂-VA score was 2 [1; 4]. Median left atrial volume index was 38 [28; 48] mL/m² and median left ventricular ejection fraction 55 [54; 58] %. See Table 1; Fig. 1.

**Table 1:** Baseline characteristics of the study population. Values are presented as median [Q1;Q3] or number (percentage), as appropriate. *AAD* antiarrhythmic drug; *AF* atrial fibrillation; *BMI* body mass index; *LAVI* left atrial volume index; *LVEF* left ventricular ejection fraction; *OAC* oral anticoagulation; *OSAS* obstructive sleep apnea syndrome; *TIA* transient ischemic attack

Variable	Value
Female, n (%)	41 (41)
Age (y)	68.0 [61.8; 76.0]
BMI (kg/m^2)^	27.2 [24.8; 29.3]
AF	
Paroxysmal, n (%)	47 (47)
Non-Paroxysmal, n (%)	53 (53)
Typical atrial Flutter, n (%)	3 (3)
CHA_2_DS_2_VA	2.0 [1.0; 4.0]
Art. Hypertension, n (%)	60 (60)
Diabetes, n (%)	15 (15)
Coronary Heart Disease, n (%)	16 (16)
Heart Failure, n (%)	15 (15)
Prior TIA/Stroke, n (%)	10 (10)
OSAS, n (%)	3 (3)
Oral Anticoaculation, n (%)	85 (85)
Antiarrhythmic Drug, n (%)	10 (10)
LVEF (%)	55.0 [54.0; 57.8]
LAVI (mL/ml)	38.0 [28.4; 48.2]
NTproBNP (ng/L)	331.0 [120.0; 977.5]
Creatinine (µmol/L)	79.9 [70.6; 99.0]

### Procedural characteristics

**Fig. 2:**
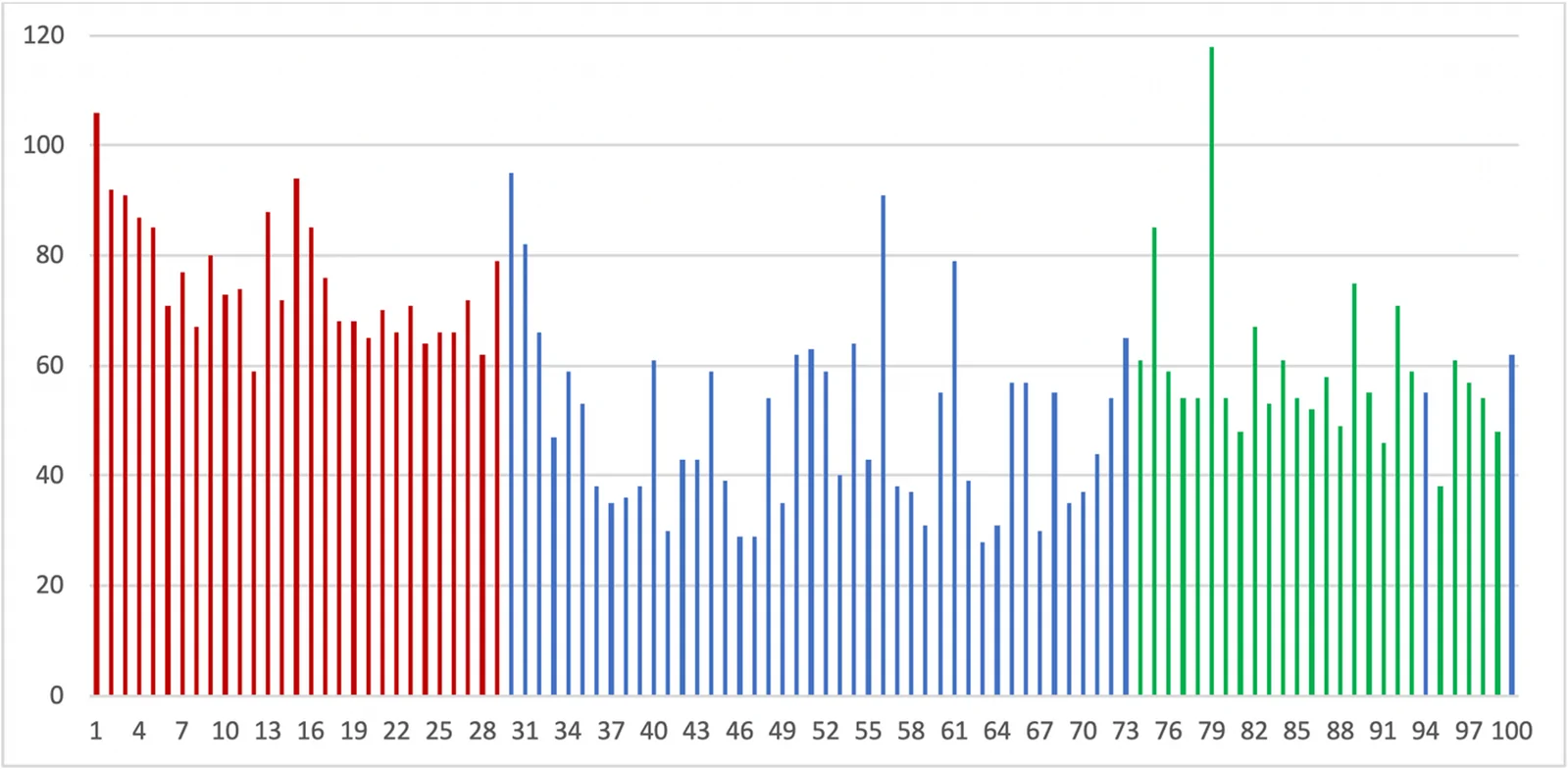
Procedure duration across the first 100 cases. Individual procedure durations for all 100 patients. Bars represent procedure time in minutes for each consecutive case. Patients enrolled in the VOLT^TM^-CE-study are shown in red, non-study patients in blue, and patients included in the VOLT^TM^-CE-extended-study cohort in green

AF was present at procedure start in 43% of patients. Deep sedation was used in 62%, and conscious sedation in 38%. Median procedure duration was 59 [47; 71] minutes, and BiB left-atrial dwell time 26 [21; 38] minutes. For PVI, the median number of applications was 4.0 for all veins (LSPV, LIPV, RIPV, RSPV). Reduced-energy delivery was required in 16% of RIPV and 13% of RSPV cases. Additional ablation was performed as follows: PWA in 41 patients, AL ablation in 3, CTI ablation in 3, and SVC isolation in 6. Across all ablation targets, acute block or isolation was achieved in 100% of cases. No intraprocedural complications occurred. One major complication was observed: a femoral pseudoaneurysm requiring interventional treatment. No additional postprocedural complications were observed during follow-up. See Tables 2 and 3; Fig. 2.

**Table 2:** Procedural characteristics of the study population. Values are presented as median [Q1;Q3] or number (percentage), as appropriate. *AF* atrial fibrillation; *GA* general anesthesia; *LA* left atrium; *PV* pulmonary vein

Variable	Value
De-novo ablation, n (%)	99 (99)
AF at procedure, n (%)	43 (43)
Sedation regime, n (%)	
Conscious	38 (38)
Deep	62 (62)
GA	0 (0)
Map, n (%)	
VOLT™	68 (68)
Mapping catheter	30 (30)
Both	1 (1)
Procedure duration (min)	59.0 [46.8; 71.0]
VOLT™ LA dwell time (min)	26.0 [21.0; 37.8]
Flouroscopy time (min)	8.2 [6.1; 10.6]
PV anatomy, n (%)	
Normal	97 (97)
Common Left	3 (3)
Common Right	0 (0)
Contrast media (mL)	40 [40; 50]
Intraprocedural cardioversion, n (%)	39 (39)

**Table 3:** Application characteristics across all ablation targets. Values are presented as median [Q1;Q3] or number (percentage), as appropriate. *AL* anterior line; *CTI* cavotricuspid isthmus; *LIPV* left inferior pulmonary vein; *LSPV* left superior pulmonary vein; *RIPV* right inferior pulmonary vein; *RSPV* right superior pulmonary vein; *PW* posterior wall; *SVC* superior vena cava

Variable	Value
LSPV	
Number of applications	4.0 [3.0; 4.0]
Reduced energy, n (%)	0 (0)
LIPV	
Number of applications	4.0 [3.0; 4.0]
Reduced energy, n (%)	0 (0)
RIPV	
Number of applications	4.0 [3.0; 4.0]
Reduced energy, n (%)	16 (16)
RSPV	
Number of applications	4.0 [2.8; 4.0]
Reduced energy, n (%)	13 (13)
Posterior wall; *n* = 41	
Number of applications	5.0 [4.0; 5.0]
Reduced energy, n (%)	0 (0)
PW acute block, n (%)	41 (100)
Number of deselected splines	3.0 [3.0; 4.0]
Anterior Line; *n* = 3	
Number of applications	7.0 [6.0; 9.0]
Reduced energy, n (%)	0 (0)
AL acute block, n (%)	3 (100)
Number of deselected splines	3.0 [3.0; 4.0]
CTI; *n* = 3	
Number of applications	5.0 [3.0; 5.0]
Reduced energy, n (%)	0 (0.0)
CTI acute block, n (%)	3 (100)
Number of deselected splines	3.0 [2.5; 4.0]
SVC; *n* = 6	
Number of applications	2.5 [2.0; 3.0]
Reduced energy, n (%)	0 (0)
SVC acute block, n (%)	6 (100)
Splines deselected, n (%)	0
Venous closure system, n (%)	26 (26)

### Subgroup analysis ablation metrics: VOLT™-CE-mark-study vs. non-study PVI-only patients

Among 57 patients undergoing PVI-only ablation, 30 (52.6%) were treated within the VOLT^TM^-CE-Mark-study (patient number 1–30), and 27 (47.4%) outside the study protocol. AF at procedure start was more frequent in the VOLT^TM^-CE-Mark-study-group (50.0% vs. 22.2%, *p* = 0.030).

Procedure duration was longer in VOLT^TM^-CE-Mark-study patients (72.5 [67.3; 85.0] vs. 48.5 [36.5; 59.0] minutes, *p* < 0.001), as was left-atrial dwell time (61.5 [56.3; 69.8] vs. 31.5 [21.5; 45.8] minutes, *p* < 0.001). BiB-specific left-atrial dwell time was similar between groups (*p* = 0.831).

Fluoroscopy time showed a trend toward lower exposure in non-VOLT^TM^-CE-Mark-study cases (*p* = 0.096). Contrast usage was higher in VOLT^TM^-CE-Mark patients (50 [42.5; 50] vs. 40 [40; 50] mL, *p* < 0.001). See Tables 4 and 5.

**Table 4:** Procedural Metrics: VOLT™-CE-Mark-Study vs. Non-Study PVI-Only Patients. Values are presented as median [Q1;Q3] or number (percentage), as appropriate. *AF* atrial fibrillation; *BiB* Balloon-in-basket; *LA* left atrium

Variable	VOLT^TM−^CE-Mark-Study	No-VOLT^TM−^CE Study	*p*-value
Number of patients, n (%)	30 (52.6)	27 (47.4)	
AF at procedure, n (%)	15 (50.0)	6 (22.2)	0.030
Procedure duration (min)	72.5 [67.3; 85.0]	48.5 [36.5; 59.0]	< 0.001
LA dwell time (min)	61.5 [56.3; 69.8]	31.5 [21.5; 45.8]	< 0.001
BiB LA dwell time (min)	25.0 [21.0; 28.2]	23.5 [18.3; 36.3]	0.831
Fluoroscopy time (min)	9.8 [7.2; 11.6]	7.6 [5.5; 9.3]	0.096
Contrast media (mL)	50.0 [42.5; 50.0]	40.0 [40.0; 50.0]	< 0.001

### Subgroup analysis ablation metrics: VOLT™-CE-mark-study vs. VOLT™-CE-mark-extended-study

Procedural parametrics differed between VOLT™-CE-Mark-study patients (*n* = 30) and those in the VOLT™-CE-Mark-Extended-study (*n* = 25). AF at procedure start was more frequent in the VOLT™-CE-Mark-study group (50.0% vs. 24.0%; *p* = 0.048). Procedure duration (72.5 [67.3–85.0] vs. 55.0 [54.0–61.0] min; *p* < 0.001) and LA dwell time (61.5 [56.3–69.8] vs. 46.0 [44.0–50.0] min; *p* < 0.001) were longer in the VOLT™-CE-Mark-study group. BiB-specific LA dwell time was higher in the VOLT™-CE-Mark-Extended-study group (40.0 [37.0–45.0] vs. 25.0 [21.0–28.2] min; *p* < 0.001) Fluoroscopy times were comparable (*p* = 0.767). Contrast use was greater in the VOLT™-CE-Mark-study group (50 [42.5–50] vs. 40 [30–40] mL; *p* < 0.001). See Table 5

**Table 5:** Procedural Metrics: VOLT™-CE-Mark-Study vs. VOLT™-CE-Mark-Extended-Study. Values are presented as median [Q1;Q3] or number (percentage), as appropriate. *AF* atrial fibrillation; *BiB* Balloon-in-basket; *LA* left atrium

Variable	VOLT^TM−^CE-Mark-Study	VOLT^TM−^CE-Mark-Extrended-Study	*p*-value
Number of patients, n (%)	30 (55.6)	25 (45.4)	
AF at procedure, n (%)	15 (50.0)	6 (24)	0.048
Procedure duration (min)	72.5 [67.3; 85.0]	55.0 [54.0; 61.0]	< 0.001
LA dwell time (min)	61.5 [56.3; 69.8]	46.0 [44.0; 50.0]	< 0.001
BiB LA dwell time (min)	25.0 [21.0; 28.2]	40.0 [37.0; 45.0]	< 0.001
Fluoroscopy time (min)	9.8 [7.2; 11.6]	9.1 [6.9; 10.7]	0.767
Contrast media (mL)	50.0 [42.5; 50.0]	40.0 [30.0; 40.0]	< 0.001

### Subgroup analysis sedation regime

A subgroup analysis was performed in patients undergoing non-study protocol PVI-only to compare procedural characteristics between deep sedation (*n* = 15, 55.6%) and conscious sedation (*n* = 12, 44.4%). Procedural metrics did not differ significantly between both sedation strategies. See Table 6.

**Table 6:** subgroup analysis comparing sedation regime. Values are presented as median [Q1;Q3] or number (percentage), as appropriate. *AF* atrial fibrillation; *BiB* Balloon-in-basket; *LA* left atrium

Variable	Deep sedation	Conscious sedation	*p*-value
Number of patients, n (%)	15 (56.6)	12 (44.4)	
AF at procedure, n (%)	3 (20)	3 (25)	0.756
Procedure duration (min)	48.0 [37.5; 60.0]	46.5 [33.5; 55.5]	0.379
LA dwell time (min)	36.0 [24.0; 44.0]	29.5 [20.8; 46.3]	0.680
BiB LA dwell time (min)	24.5 [21.3; 33.0]	21.5 [17.8; 37.5]	0.520
Fluoroscopy time (min)	8.7 [6.0; 9.3]	7.0 [5.3; 8.8]	0.427
Contrast media (mL)	40.0 [40.0; 45.0]	40.0 [40.0; 40.0]	0.375

### Biomarker response

Changes in biomarker levels between baseline and post-procedural measurements are summarized by the corresponding delta values. Leukocytes showed a Δ of 1.1 [−0.1; 3.0] ×10⁹/L, CRP 3.3 [1.1; 6.1] mg/L, LDH 52.0 [31.5; 68.5] U/L, myoglobin 13.0 [0.0; 30.0] ng/mL, CK 197.0 [123.0; 287.5] U/L, and troponin T 1096.8 [826.5; 1672.0] ng/L. Hemoglobin Δ − 0.4 [−1.1; 0.4] g/dL, platelets Δ − 7.5 [−21.8; 4.3] ×10⁹/L, haptoglobin Δ − 0.03 [−0.14; 0.05] g/L, bilirubin Δ 2.4 [0.0; 7.4] µmol/L, creatinine Δ 4.0 [−3.0; 11.5] µmol/L, and GFR Δ − 2.5 [−9.8; 2.8] mL/min.

No significant correlations were observed between the number of applications and changes in any biomarker. See Tables 7 and 8.

**Table 7:** Comparison of pre- and post-procedural biomarkers Values are presented as median [Q1; Q3]. *CK* creatine kinase; *CRP *C-reactive protein; *eGFR *estimated glomerular filtration rate; *LDH *lactate dehydrogenase

	Timing		*p*-value
Leukocytes (*10^9/L)	Pre	6.1 [5.2; 7.5]	<.001
	Post	7.8 [6.6; 9.4]	
Platelets (*10^9/L)	Pre	209.0 [175.0; 250.0]	<.001
	Post	195.5 [161.8; 240.3]	
Hemoglobin (g/dL)	Pre	13.6 [12.3; 14.7]	0.001
	Post	13.3 [12.2; 14.2]	
CRP (mg/l)	Pre	1.2 [0.6; 2.2]	<.001
	Post	6.0 [3.2; 9.5]	
Haptoglobulin (g/L)	Pre	1.1 [0.8; 1.4]	0.422
	Post	1.1 [0.8; 1.4]	
Bilirubin (µmol/L)	Pre	10.9 [8.2; 13.9]	0.132
	Post	13.8 [9.7; 19.1]	
LDH (U/L)	Pre	178.0 [161.0; 199.0]	<.001
	Post	238.0 [204.3; 263.8]	
Creatinine (µmol/L)	Pre	79.9 [70.6; 99.0]	<.001
	Post	86.0 [75.0; 99.3]	
GFR (mL/min)	Pre	76.0 [63.0; 91.0]	<.001
	Post	77.0 [60.0; 89.0]	
Myoglobin (ng/mL)	Pre	44.0 [36.0; 58.0]	<.001
	Post	52.5 [46.0; 77.3]	
CK (U/L)	Pre	85.0 [61.8; 121.0]	<.001
	Post	306.0 [217.0; 405.0]	
Troponin-T (ng/L)	Pre	9.5 [6.2; 13.0]	<.001
	Post	1077.0 [746.0; 1549.0]	

**Table 8:** Correlation between energy applications and biomarker changes. *CK* creatine kinase; *CRP* C-reactive protein; *eGFR* estimated glomerular filtration rate; *LDH* lactate dehydrogenase

Marker		*p*-value
Leucocytes	−0.050	0.649
Hemoglobin	0.003	0.979
Platelets	0.017	0.879
CRP	−0.014	0.902
Haptoglobin	−0.198	0.092
Billirubin	−0.058	0.621
LDH	0.165	0.156
Creatinine	0.110	0.324
GFR	−0.059	0.600
Myoglobin	−0.144	0.223
CK	−0.039	0.740
Troponin T	0.033	0.785

### Follow-up

**Fig. 3:**
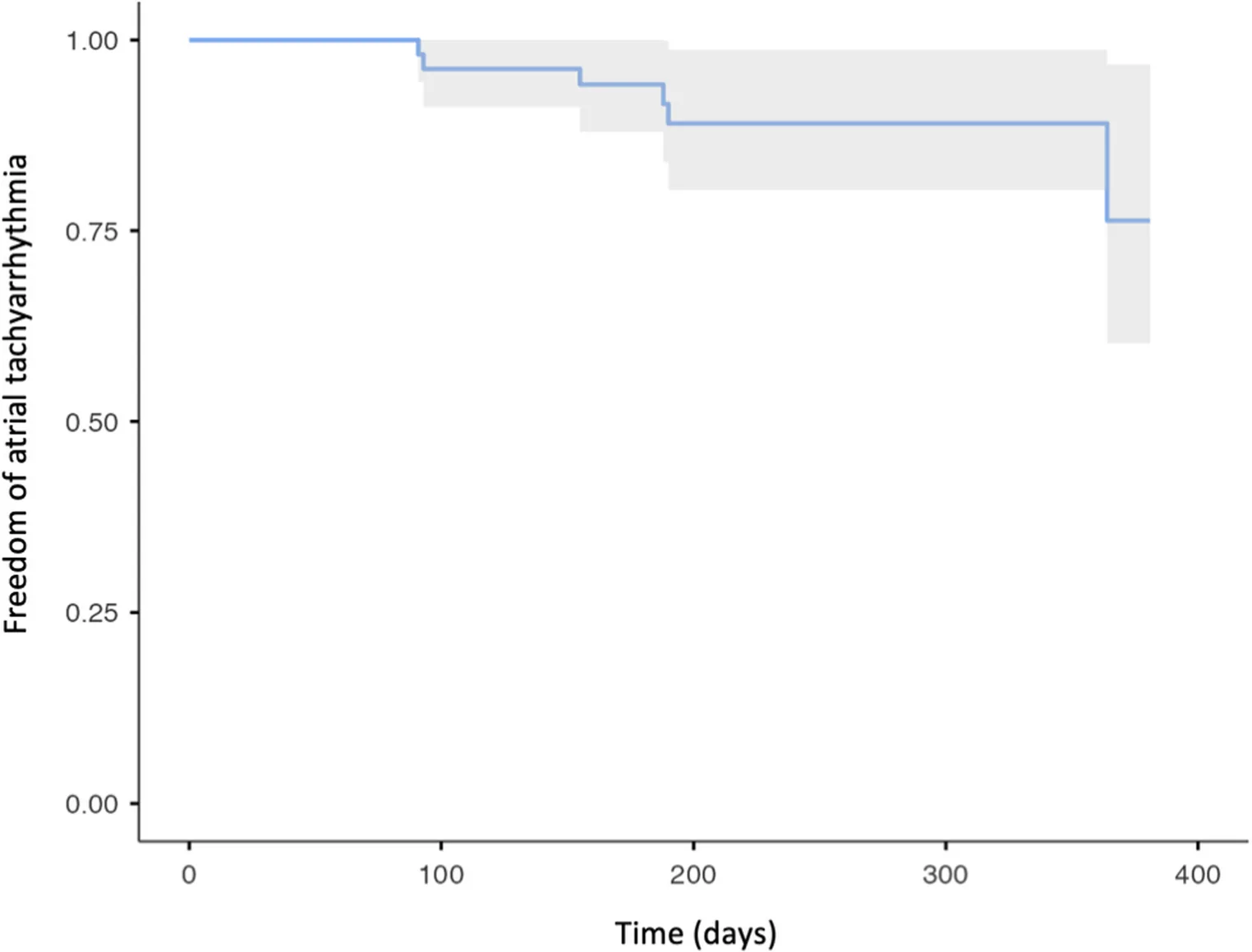
Kaplan–Meier estimate of arrhythmia-free survival at 12 months. Any documented atrial tachyarrhythmiaon ECG, Holter monitoring, or trans-telephonic monitoring after the blanking period was considered an event

Follow-up of at least 6 months was available in the first 52 patients, corresponding to all patients enrolled until June 2025. Among patients with follow-up, 50% had persistent AF. After a 60-day blanking period, freedom from atrial tachyarrhythmia was 86% at a median follow-up of 293 [184–364] days. Arrhythmic recurrence was AF in 71.4%, atrial tachykardia in 28.6%. Median time to recurrence was 188 [123–277] days. Five patients underwent additional posterior wall ablation, none of whom experienced arrhythmic recurrence. See Fig. 3.

## Discussion

This observational prospective study is the first to systematically assess the procedural performance and acute safety profile of PFA using the BiB-PFA-system in an unselected cohort treated at a high-volume electrophysiology center. While previous investigations of this technology have primarily been conducted exclusively within controlled study settings, evidence from routine clinical practice has been lacking.

The present data support the feasibility, workflow stability, and periprocedural safety of the BiB-PFA-system across a broad AF population. Key observations from this cohort include:

- Consistently effective lesion delivery, with 100% acute PVI success and achievement of acute block/isolation at additional ablation targets.
- Freedom from atrial tachyarrhythmia of 86% after a 60-day blanking period at a median follow-up of 293 [184–364] days in a subset of patients, suggesting favorable early rhythm outcomes.
- Efficient procedural workflows, reflected by short procedure and LA dwell times, even when extended ablation (PWA, AL, CTI, SVC) was performed.
- A favourable safety profile, with low rate of periprocedural complications, including one vascular complication requiring intervention.
- Protocol-driven workflow differences between VOLT-CE-Mark-study and non-study PVI-only patients, mainly attributable to additional mapping requirements in the study cohort.

### Safety

In this cohort, the BiB-PFA-system demonstrated a favorable acute safety profile, with only one major postprocedural vascular event requiring intervention. Importantly, no PFA-related adverse events were observed, including phrenic nerve injury, clinical apparent esophageal injury, coronary vasospasm, or clinically significant hemolysis. These results closely mirror the safety outcomes previously reported in the VOLT-CE-Mark and VOLT-AF-IDE-studies, further supporting the consistency of the BiB-PFA-system’s acute safety performance across controlled and clinical settings [2, 6]

This contrasts with using pentaspline PFA-catheters and with cryoballoon ablation, in which phrenic nerve palsy remains a relevant risk [11, 12]. The balloon-based nature of the BiB- catheter—and operator familiarity with balloon navigation from cryoablation workflows—may contribute to lower mechanical complication rates and a shorter learning curve.

The design characteristics of the BiB-PFA-system may contribute to this favourable profile. Stable tissue contact, integrated contact feedback, and reduced electrode exposure to the blood pool help minimise shear forces and intravascular energy delivery—factors previously associated with hemolysis in other PFA-systems. Correspondingly, no clinically significant hemolysis or acute kidney injury occurred in this cohort. In the present cohort, which included both PVI-only procedures and cases with additional lesion sets, biomarker analysis showed a measurable inflammatory response and marked myocardial biomarker release, while hemolysis-related biomarkers showed only modest changes. These findings are in line with previous investigations from our working group, in which the biomarker profile of the BiB-system was characterized by low inflammatory and hemolytic activation alongside pronounced troponin release, potentially suggesting effective myocardial electroporation [3–5]. These mechanistic insights align with the system’s architecture, including selective spline deactivation for additional lesion sets, which limits blood-pool energy delivery during non-PV ablation.

Moreover, biomarker profiles from mechanistic studies by our group have demonstrated reduced inflammatory response and hemolysis compared with both thermal energy sources and pentaspline-based PFA, supporting a favorable safety profile from a biomarker perspective [3–5].

Taken together, the findings confirm that the BiB-PFA-system can be deployed safely in contemporary clinical practice, with a safety profile that is consistent across study-driven and real-world treatment environments.

### Efficacy

Acute procedural effectiveness was high, with 100% isolation of all pulmonary veins and complete acute block across all extended ablation targets, including posterior wall, anterior line, CTI, and SVC. These results are consistent with acute success rates reported for other PFA-systems [13, 14] and suggest that the integration of mapping and ablation in a single catheter does not compromise lesion formation.

The ability to target non-PV structures efficiently reflects the flexibility of selective spline activation. In this study, additional lesion sets could be completed without catheter exchange or complex manoeuvring, which may help reduce variability in lesion quality. Procedural parameters such as the number of applications per vein showed low variability, indicating stable catheter–tissue interaction and predictable lesion delivery. Although limited to a subset of patients, the observed 86% freedom from atrial tachyarrhythmia after a 60-day blanking period at mid-term follow-up is consistent with early outcome data reported for contemporary PFA technologies [15]. Overall, the acute effectiveness observed in this cohort confirms that the BiB-PFA system provides fast and reliable ablation across a broad range of atrial targets.

### Clinical Implications

The present study provides important insight into how the BiB-PFA system integrates into routine electrophysiology workflows. Procedural efficiency was high, with short procedure and left atrial dwell times—even when additional ablation beyond PVI was performed. A key contributor to this efficiency is the system’s integrated mapping capability, which enabled complete anatomical reconstruction without the need for a supplementary mapping catheter in all non-study patients. The subgroup comparisons further illustrate these findings: while procedures performed under the VOLT-CE-Mark study required a dedicated mapping catheter and consequently showed longer left-atrial dwell and procedure times, non-study cases using mapping-only-with-BiB demonstrated time savings, highlighting the practical workflow advantages of the integrated approach. The marked reduction in procedure time observed after the initial study cases reflects a rapid learning curve and efficient operator adaptation to the integrated BiB-PFA workflow.

Additional procedural simplifications arise from the catheter design. The BiB-PFA system requires only a standard SL1 (Abbott) wire for pulmonary vein access, avoiding the need for a 0.035-inch extra-support guidewire used with other PFA systems. Although the SL1 wire must be reintroduced after sheath exchange, this approach reduces equipment handling and material exchange. Furthermore, atrial anatomy and posterior wall geometry can be assessed during a “fly-by” manoeuvre, for example when transitioning from the LIPV to the RSPV, eliminating the need for dedicated mapping passes or catheter exchanges.

Sedation flexibility represents an important clinical aspect. In this cohort, procedures were successfully performed under both deep and conscious sedation without compromising catheter stability or lesion delivery. Unlike other PFA technologies that often require deeper sedation due to painful energy delivery, the tolerability of this system under conscious sedation supports leaner and potentially more ambulatory workflows. This is particularly relevant in health systems where propofol administration requires anaesthesia support or where same-day discharge pathways are increasingly adopted. Notably, an exploratory subgroup analysis in PVI-only patients showed comparable procedural efficiency between conscious and deep sedation, with a numerical trend toward shorter procedure duration and reduced LA-dwell time under conscious sedation. These observations are further supported by our previously published work, which demonstrated high patient satisfaction and a favourable safety profile of PFA procedures performed under conscious sedation. ^8^ However, this analysis is based on a limited number of patients and should therefore be interpreted with caution, without allowing conclusions regarding equivalence between sedation strategies.

Taken together, these findings suggest that the BiB-PFA system can be integrated into high-volume electrophysiology practice, with potential implications for procedural workflow and resource utilization across both PVI and extended ablation strategies. Future multicentre studies will be important to confirm whether these procedural advantages translate into measurable clinical and economic benefits.

### Limitations

This study has several limitations. First, it represents a single-centre experience from a high-volume electrophysiology centre, which may limit generalisability to other settings with different workflows, operator experience, or case mixes. Second, although data collection was prospective, the analysis was observational and non-randomised, precluding causal interferences or comparisons with alternative ablation modalities. Third, the overall sample size, particularly for extended ablation strategies such as anterior line, CTI, or SVC ablation, remains modest.

Fourth, acute success was assessed using standard remapping and pacing manoeuvres, while invasive durability testing was not systematically evaluated. Fifth, procedural differences between VOLT-CE-Mark-study and non-study cases were partly driven by study-mandated mapping requirements and may also reflect evolving operator experience.

Sixth, subgroup comparisons may have been influenced by a learning curve effect. Seventh, biomarker analyses were performed in a cohort including both PVI-only procedures and cases with additional lesion sets beyond PVI. Eighth, follow-up beyond 6 months was available only in a subset of patients, limiting the interpretation of longer-term rhythm outcomes. However, atrial arrhythmia recurrences occurring beyond 6 months are relatively uncommon, and a 6-month follow-up is increasingly used as a primary endpoint to assess longer-term efficacy in contemporary AF ablation studies [2]. Nineth, rhythm monitoring was limited to scheduled 24-hour Holter recordings rather than continuous monitoring or implantable devices, which likely resulted in underdetection of asymptomatic recurrences. Furthermore, arrhythmia recurrences were not stratified according to baseline AF type (paroxysmal versus persistent), and outcomes were therefore reported for the overall cohort. In addition, repeat ablation procedures were not included in the predefined analytical scope of this study and are going to be addressed in a dedicated follow-up analysis. Tenth, two patients were excluded from the present analysis, including one case with markedly prolonged procedural duration due to a non-procedural delay and one case of pericardial tamponade occurring prior to energy delivery, related to transseptal puncture. Inclusion of these cases would have influenced procedural metrics without reflecting device-related performance. Exclusion of these cases may therefore introduce bias. Eleventh, the use of additional lesion sets beyond PVI introduces procedural heterogeneity, which may influence procedural characteristics and clinical outcomes and should be considered when interpreting the results. Finally, as a pioneer centre using the BiB-PFA-system, international visitors and live-case demonstrations were frequently hosted during the study period, which may have introduced procedural pauses or modifications and influenced workflow metrics.

## Conclusion

This initial high volume single-center evaluation of the BiB-PFA-system demonstrates that the technology delivers rapid, reliable, and versatile lesion formation across pulmonary veins and extended atrial targets with a 100% acute success rate. The ability to combine mapping and ablation within a single catheter, together with stable performance under both deep and conscious sedation, highlights the system’s potential to streamline workflows and reduce procedural complexity. The favourable acute safety profile observed in an unselected clinical cohort underscores the maturity of this PFA approach and supports its integration into routine AF ablation practice. In a subset of patients with available follow-up, a high rate of freedom from atrial tachyarrhythmia after the blanking period was observed, indicating favorable early rhythm outcomes. These findings position the BiB-PFA-system as a promising platform for efficient, adaptable, and tissue-selective ablation, warranting further multicentre investigations to confirm durability and long-term clinical outcomes.

## Data Availability

Data supporting this study are curated at the Study Center of the Department of Rhythmology, University Hospital SchleswigHolstein, Germany.
